# Interference of internal waves due to two point vortices: linear analytical solution and nonlinear interaction

**DOI:** 10.1098/rsos.211476

**Published:** 2022-04-13

**Authors:** Zhen Wang, Di Liu, Xiaoqin An

**Affiliations:** School of Mathematical Sciences, Dalian University of Technology, Dalian, 116024, People's Republic of China

**Keywords:** stratified flow, point vortex pairs, boundary integral equations, linearized solution, wave interference, nonlinear interfacial waves

## Abstract

In this work, we consider steady two-dimensional interfacial waves in a two-layer stratified fluid, which is induced by a vortex pair located in the lower layer of the fluids. A mathematical model based on the boundary integral equation method and the potential-flow theory is established. The linear analytical solution for the linearized model is given in the form of Cauchy integral and then asymptotic behaviour for large x is presented. The fully nonlinear model is solved by the Jacobian-free Newton–Krylov (JFNK) method numerically. Nonlinear characteristics of wave profiles are identified compared with the linear results under different vortex strengths and the distance between the vortex pair. The amplitude of steady downstream waves is found to vary periodically with respect to the distance of the vortex pair, which can be regarded as the interference between waves produced by each vortex. For equal-strength counter- and co-rotating pairs, the downstream wave heights of linear solutions can be eliminated for some special values of the distance between point vortices, namely, the destructive interference occurs. Meanwhile, the wave only exists between the vortex pair like trapped waves. So does the nonlinear counterpart for counter-rotating pairs, but it could not be diminished with any distance.

## 1. Introduction

Water stratification is often encountered in specific ocean areas with glacier melting or river injection. On the interface of two adjacent layers of the stratified fluid, internal waves can be caused by disturbances [1]. It is recognized that internal waves may have close relationships with many offshore human activities. For example, engineers have considered internal waves as an important threat to offshore drilling operations, the strength of deep-water risers, or navigation of underwater vehicles [2]. Besides, ocean internal waves play a significant role in ocean mixing processes, energy transporting, redistributing nutrients and heat, driving the circulations of climate factors like heat and carbon, and so on [3–6]. The generation of internal waves includes various complex mechanisms, which is still an ongoing research subject [7–10].

Many researchers have proposed various generation mechanisms of internal waves. A simple two-layer model of a stratified fluid is frequently employed. Yeung & Nguyen [11] investigated internal waves generated by a moving point source in the upper layer of a finite-depth stratified fluid. The Green function method was applied to analyse wave patterns of the interface. Then Wei *et al.* [12] studied the case of a moving point source in the lower layer and compared the model with experimental results in [13]. Wei *et al.* [14] also considered the case of internal waves generated by a moving dipole in the lower layer, where both analytical and experimental results were discussed. Moreover, for the two-dimensional ideal flows over a semicircular obstruction, Forbes & Schwartz [15] used the Cauchy integral formula to derive an integro-differential equation coupled with Bernoulli's equation with respect to the potential function. Subsequently, Forbes proposed an approach of using the arclength parameter to describe the free surface and construct boundary integral equations, and applied this approach to study nonlinear surface waves caused by a submerged point vortex [16] and a submerged hydrofoil [17]. Via this boundary integral method, for nonlinear internal waves caused by the vortex, Wang *et al.* [18,19] discussed internal waves generated by a point vortex in either the lower or the upper layer of an infinite stratified liquid, respectively. Strong nonlinear waves were observed in the case of a submerged point vortex in the lower layer. When the absolute value of negative vortex circulation is large, the whole wave profile is similar to a solitary wave. Besides, Wang *et al.* [20] studied internal waves generated by a hydrofoil in the lower layer and analysed the influence on the wave profile of different parameters of background flows and the size of the hydrofoil. They found that the amplitude of interfacial waves increases firstly and then decreased as the location of the hydrofoil rises vertically. With the assumption that the upper-layer fluids flow under a flexible elastic sheet, Wang *et al.* [21] used the boundary integral equation method with respect to the potential function to calculate the interfacial solitary waves. Besides, the above works all focused on the case of steady wave solution. Boundary integral equation method (BIEM) have been verified to be an efficient method for these cases. Ambrose [22] proved the well-posedness of two-dimensional ideal irrotational fluids described by boundary integral equations. Especially, Ambrose confirmed that the time-invariant solution to this problem is stable.

When two submerged disturbances exist, trapped waves may occur. As a special kind of critical flows, waves are 'trapped' and only maintained between two obstructions. In linearized problems subcritical waveless solutions can be derived by superposition of multiple solutions namely appropriate combinations of obstructions can lead to the trapped waves, while it may be not completely true for nonlinear problems. From weakly nonlinear theory, Dias & Vanden-Broeck [23] used the Korteweg–De Vries (KdV) theory as well as nonlinear numerical computations and found the generalized hydraulic falls of free-surface flows past two obstructions. Waves are trapped between two obstructions and the surface has a significant fall after the second obstruction. Holmes *et al.* [24] used fully nonlinear methods and obtained various subcritical waveless flows past two symmetric bottom topographies with different solution behaviours. An elaborate description of solution branches in the parameter with respect to the heights and the separation distance of two topographies was provided, in which every point on the branch represents a waveless solution. Then Holmes & Hocking [25] used KdV theory to re-examine this problem and compared the results from nonlinear methods, KdV theory and linearized methods. Besides, contours in parameter space of separation distances and heights of obstructions are depicted under different widths of the obstruction and Froude numbers. When surface tensions are considered, Page *et al.* [26] studied this critical solution of steady two-dimensional gravity-capillary forced free-surface flow past submerged obstructions. Employing boundary integral equations, different surface tensions hydraulic falls were derived and new types of trapped waves between two obstructions were found upstream/downstream of the hydraulic fall. Besides, flows over topographies may also lead to waves trapping, reflecting, focusing and resonance [27]. Using tools from dynamical systems theory and Green function techniques, Echeverri *et al.* [28] demonstrated the internal tide attractors theoretically and experimentally, where waves scattered from multiple ridges and trapped within them. Moreover, in the absence of surface tensions, Vanden-Broeck [29] computed multiple particular trapped waves under the surface pressure distributions, which can simulate objects moving on/under the surface at a constant velocity. These waveless solutions are related to the design of drag-free objects. In this work, we extend the works in [18] and infer that trapped waves due to two vortices may occur in certain cases.

In irrotational ideal fluids, the point vortex can model the motion of a moving body in the zero-radius limit [30]. Thus the problem of multiple moving bodies could be studied via a point vortex system inspired by the findings of waveless solutions in the case of two topographies. And the constructive/destructive interference between the interfacial waves may occur in this case. Besides, a pair of vortices is also a simple model of a moving body whose size is modelled by the separation distance of the pair. On the other hand, vortex pairs often generate in the wake of moving bodies, e.g. aircraft wings [31]. We expect it may improve our understanding of complex flows, especially in strong stratified fluids, to study the flows due to a pair of vortices.

The structure of this paper is as follows: in §2, integral-differential equations are established using the potential flow theory and boundary integral equation method (BIEM). In §3, the linearized solution is obtained. And the asymptotic behaviour of this solution for large *x* can also be obtained. Meanwhile, the model is also solved by the Jacobian-free Newton–Krylov (JFNK) method. In §4, we discuss the differences between nonlinear and linear solutions for both counter- and co-rotating pairs. Effects of the distance between two vortices and vortex strength are also considered. The conclusion is given in §5.

## 2. Model of the problem

The case of steady two-layer fluids of different densities is considered. Both layers are assumed to be ideal and steady fluids. The uniform velocities are set in upstream. A Cartesian coordinate system is created such that the *x*-axis is placed along the undisturbed horizontal interface pointing in the same direction of upstream uniform flow, as well as the *y*-axis points up vertically. The depth of the upper fluid is *T* and its upper surface with the rigid-lid assumption. The lower fluid is infinitely deep, where two point vortices are placed at (−*D*, −*H*) and (*D*, −*H*), the centre of the vortex pair located at (0, −*H*). The circulations of two point vortices are *K*_1_ and *K*_2_, respectively. In the following context, we use subscripts 1 and 2 to represent other physical variables associated with the upper and lower fluids, respectively. The densities of the two layers are *ρ*_1_, *ρ*_2_. The upstream uniform velocities of the two layers are *γ*_1_ and *γ*_2_.

**Figure 1.**
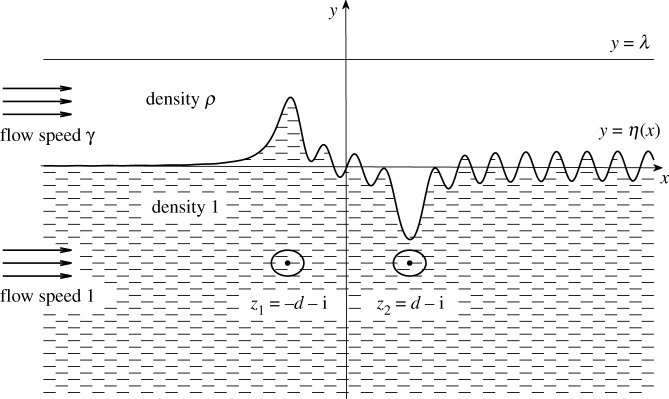
The dimensionless model of a two-layer flow about a submerged vortex pair located horizontally.

For the convenience of discussion, we derive the dimensionless model based on the reference length *H* and the reference velocity *γ*_2_. The following dimensionless parameters are introduced:

$$\begin{array}{rl} & F=\frac{\gamma_{2}}{\sqrt{gH}},\;\epsilon_{1}=\frac{K_{1}}{\gamma_{2}H},\;\epsilon_{2}=\frac{K_{2}}{\gamma_{2}H},\\ & \;\rho =\frac{\rho_{1}}{\rho_{2}},\;\gamma =\frac{\gamma_{1}}{\gamma_{2}},\;\lambda =\frac{T}{H},\;d=\frac{D}{H}, \end{array}$$

where *F* is the Froude number, *ϵ*_1_ and *ϵ*_2_ are the dimensionless vortex strength of two vortices, *ρ* is the density ratio, *γ* is the far upstream uniform velocity ratio, *λ* is the dimensionless depth of the upper layer and 2*d* is the dimensionless distance between two vortices. The elevation of the fluid interface is described by *y* = *η*(*x*). Figure 1 gives the schematic diagram of this problem and its settings.

Potential functions *ϕ*_1_, *ϕ*_2_, stream functions *ψ*_1_, *ψ*_2_ satisfy the Cauchy–Riemann equations from potential theory,

2.1 $$\frac{\partial \phi_{j}}{\partial x}=\frac{\partial \psi_{j}}{\partial y},\;\frac{\partial \phi_{j}}{\partial y}=-\frac{\partial \psi_{j}}{\partial x},\;j=1,2.$$

Then complex potentials *f*_*j*_(*z*) = *ϕ*_*j*_(*x*, *y*) + i*ψ*_*j*_(*x*, *y*) (*j* = 1, 2) in terms of *z* = *x* + i*y* are introduced for upper and lower fluids separately. Both functions are analytic except for the position of the two vortices *z*_1_ = −*d* − i, *z*_2_ = *d* − i, where i^2^ = −1. *f*_2_ satisfies

2.2 $$f_{2}\to z+\sum_{\;j=1}^{2}\frac{i\epsilon_{j}}{2\pi }\log(z-z_{j}),\;\mathrm{as}\;z\to z_{j},\;j=1,2.$$

The upstream radiation conditions for the upper layer and lower layer as well as for the interface are

2.3 f1→γz,f2→z, η→0, Re[z]→−∞,

where Re[*z*] means the real part of *z*. The upper surface is assumed as a rigid lid. At the interface as well as the rigid upper surface the unpenetrable conditions give

2.4 $$\nabla \phi_{\;j}\cdot n=0,\;j=1,2,\;\text{on}\;y=\eta (x)$$

and

2.5 $$\frac{\partial \phi_{1}}{\partial y}=0,\;\text{on}\;y=\lambda ,$$

where ∇=(∂/∂x,∂/∂y).

Introducing the arclength parameter *s* to parametrize the fluid interface *y* = *η*(*x*), then the fluid interface is represented as (*x*, *y*) = (*x*(*s*), *y*(*s*)). The arclength condition satisfies

2.6 $${\left(\frac{dx}{ds}\right)}^{2}+{\left(\frac{dy}{ds}\right)}^{2}=1.$$

According to setting the pressure equally on the interface, the fluid interface satisfies Bernoulli's equation

2.7 $$\rho {\left(\frac{d\phi_{1}}{ds}\right)}^{2}-{\left(\frac{d\phi_{2}}{ds}\right)}^{2}+\frac{2(\rho -1)y}{F^{2}}=\rho \gamma^{2}-1.$$

This is obtained as follows [18]: for the upper-layer fluids Bernoulli's equation is

$$p_{\mathrm{upper}}+\frac{1}{2}\rho {\left(\frac{d\phi_{1}}{ds}\right)}^{2}+\frac{\rho y}{F^{2}}=\frac{1}{2}\rho \gamma^{2},$$

and for the lower-layer fluids that is

$$p_{\mathrm{lower}}+\frac{1}{2}\rho {\left(\frac{d\phi_{2}}{ds}\right)}^{2}+\frac{y}{F^{2}}=\frac{1}{2},$$

under the assumption that the pressure along the separating streamlines at infinity is zero. Thus the condition of pressure continuity, namely *p*_upper_ = *p*_lower_ along the interface, gives (2.7).

Similar to the derivation process proposed by [18], we choose a fixed point *z* = *z*(*s*) on the fluid interface, where *z*(*s*) = *x*(*s*) + i*y*(*s*), and two integral-differential equations are established for the upper and lower layer by the BIEM. For the upper layer, we introduce the analytic function

2.8 $$G_{1}(z)=\frac{df_{1}}{dz}-\gamma .$$

According to the Cauchy integral formula, we have

2.9 $$\oint_{\Gamma_{1}}\frac{G_{1}(\xi )}{\xi -z(s)}\;d\xi =\pi iG_{1}(z(s)),$$

where the integral path $\Gamma_{1}$ consists of the interface *y* = *η*(*x*) excluding the point *z* = *z*(*s*), a semicircle centring *z* = *z*(*s*) and bypassing it in the upper layer, the image of the interface *y* = 2*λ* − *η*(*x*) with respect to *y* = *λ*, two vertical lines *x* = ±*L* connecting the interface *y* = *η*(*x*) and its image *y* = 2*λ* − *η*(*x*) for sufficiently large *L*. Under the kinematic condition on the surface of the upper layer (2.5), or equally, Im*G*_1_ = 0 on *y* = *λ*, *G*_1_(*z*) can be continued analytically to its conjugate counterpart on the image region of the upper-layer domain, i.e.

$$G_{1}(x+iy)=\bar{G_{1}(x+i(2\lambda -y))}.$$

From the above discussions, it is reasonable to do the contour integration along $\Gamma_{1}$. Besides, by virtue of the asymptotic properties of *G*_1_(*z*); that is to say, *G*_1_(*z*) → 0, *x* → −∞, and |*G*_1_| < +∞, |*G*_1_(*z*)| = *o*(|*z*|), *x* → +∞, the contributions from *x* = ±*L* are both zero. Taking the imaginary part of (2.9) gives

2.10 $$\begin{array}{rl} & \pi (\gamma -x^{\prime }(s)\phi_{1}^{\prime }(s))=\int_{-\infty }^{+\infty }\frac{\left|\begin{array}{cc} x(t)-x(s) & y(t)-y(s)\\ \gamma x^{\prime }(t)-\phi_{1}^{\prime }(t) & \gamma y^{\prime }(t) \end{array}\right|}{{\left(x(t)-x(s)\right)}^{2}+{\left(y(t)-y(s)\right)}^{2}}\;dt\\ & \;+\int_{-\infty }^{+\infty }\frac{\left|\begin{array}{cc} x(t)-x(s) & y(t)+y(s)-2\lambda \\ \gamma x^{\prime }(t)-\phi_{1}^{\prime }(t) & \gamma y^{\prime }(t) \end{array}\right|}{{\left(x(t)-x(s)\right)}^{2}+{\left(y(t)+y(s)-2\lambda \right)}^{2}}\;dt. \end{array}$$

Comparing with the full form like equation (15) in [18],

$$\begin{array}{rl} & \pi (\gamma -x^{\prime }(s)\phi_{1}^{\prime }(s))=\int_{-\infty }^{+\infty }\frac{\left(\phi_{1}^{\prime }(t)-\gamma x^{\prime }(t))(y(t)-y(s))+\gamma y^{\prime }(t)(x(t)-x(s)\right)}{{\left(x(t)-x(s)\right)}^{2}+{\left(y(t)-y(s)\right)}^{2}}\;dt\\ & \;+\int_{-\infty }^{+\infty }\frac{\left(\phi_{1}^{\prime }(t)-\gamma x^{\prime }(t))(y(t)+y(s)-2\lambda )+\gamma y^{\prime }(t)(x(t)-x(s)\right)}{{\left(x(t)-x(s)\right)}^{2}+{\left(y(t)+y(s)-2\lambda \right)}^{2}}\;dt, \end{array}$$

the matrix form in (2.10) is more compact. This expression with Jacobian is more or less similar to the formulation of the Biot–Savart Law for three-dimensional vortex, though its specific physical significance may be explored in the following works.

For the lower layer, we introduce the analytic function

2.11 $$G_{2}(z)=\frac{df_{2}}{dz}-1.$$

The equation

2.12 $$\oint_{\Gamma_{2}}\frac{G_{2}(\xi )}{\xi -z(s)}\;d\xi =\pi iG_{2}(z(s))+2\pi i\sum_{k=1,2}\mathrm{Res}\left\{\frac{G_{2}(\xi )}{\xi -z(s)},z_{k}\right\},$$

is obtained based on residue theorem, where the integral path $\Gamma_{2}$ consists of the interface excluding the point *z* = *z*(*s*), a semicircle centring *z* = *z*(*s*) and bypassing it in the lower layer, a semicircle with a large radius *R* in the limiting *R* → +∞ immersed in the lower layer. Taking the imaginary part of (2.12) gives

2.13 $$\begin{array}{rl} & \pi (x^{\prime }(s)\phi_{2}^{\prime }(s)-1)=\int_{-\infty }^{+\infty }\frac{\left|\begin{array}{cc} x(t)-x(s) & y(t)-y(s)\\ x^{\prime }(t)-\phi_{2}^{\prime }(t) & y^{\prime }(t) \end{array}\right|}{{\left(x(t)-x(s)\right)}^{2}+{\left(y(t)-y(s)\right)}^{2}}\;dt\\ & \;+\frac{\epsilon_{1}(y(s)+1)}{{\left(x(s)+d\right)}^{2}+{\left(y(s)+1\right)}^{2}}+\frac{\epsilon_{2}(y(s)+1)}{{\left(x(s)-d\right)}^{2}+{\left(y(s)+1\right)}^{2}}. \end{array}$$

In (2.10) and (2.13), the first integrals, which are singular integrals, are both in the Cauchy-value sense as *t* → *s*. Equation (2.13) suggests that the only difference between the case of one vortex and that of two vortices in this paper is the last two terms in (2.13). If |*ε*_1_| = |*ε*_2_| = |*ε*| and *d* → 0, these two terms becomes

$$-\mathrm{sgn}(\epsilon_{1})\frac{4d|\epsilon |x(s)(y(s)+1)}{{\left(x^{2}(s)+{\left(y(s)+1\right)}^{2}\right)}^{2}}\;+O(d^{2})\;(\epsilon_{1}+\epsilon_{2}=0),$$

or

$$\frac{2\epsilon (y(s)+1)}{x^{2}(s)+{\left(y(s)+1\right)}^{2}}+O(d^{2})\;(\epsilon_{1}=\epsilon_{2}=\epsilon ),$$

which suggests that the case of equal-length co-rotating pair degenerates to the case of one vortex which strength is the sum of these two vortices.

The governing equations (2.6), (2.7), (2.10) and (2.13) are derived. *x*(*s*), *y*(*s*), *ϕ*_1_(*s*) and *ϕ*_2_(*s*) can be determined by numerical methods.

## 3. Methodology

### 3.1. Linear analytical solution

In this subsection, we will give the linearized solution of interfacial wave profiles for two vortices. We can use the results proposed in [18], which provides the linearized solution *ϕ*_+_, *ϕ*_−_ for one vortex at (*x*_0_, −*h*). By superposition principle, velocity potentials *ϕ*_1_ (upper layer) and *ϕ*_2_ (lower layer) for a vortex pair can be written as the sum of two velocity potentials due to two separate vortices at (±*d*, −1), since they are solutions of linearized problems. Here, we briefly review the linearized problems and do not provide the specific formulations of the velocity potentials. One can refer to [18] for their lengthy expressions.

The linearized kinematic boundary condition on the interface is

3.1 $$\frac{\partial \phi_{1}}{\partial y}=\gamma \frac{d\eta }{dx},\;\frac{\partial \phi_{2}}{\partial y}=\frac{d\eta }{dx},\;y=0,$$

and the linearized Bernoulli's equation on the interface is

3.2 $$\rho \gamma \frac{\partial \phi_{1}}{\partial x}-\frac{\partial \phi_{2}}{\partial x}+\frac{\rho -1}{F^{2}}\eta =0,\;y=0.$$

The kinematic boundary condition along the surface of the upper layer is still (2.5). Only in this subsection we use the subscripts ‘+’ and ‘−’ to present the potentials of the upper and lower layer due to one vortex, respectively. The solutions of this linearized problem *ϕ*_+_, *ϕ*_−_ due to a vortex at any position (*x*_0_, −*h*) (*h* > 0) with any strength *ε* had been derived in equations (29) and (30) from [18] in the form of integrals. Next, we set *x*_0_ = −*d*, *h* = 1, *ε* = *ε*_1_ and *x*_0_ = *d*, *h* = 1, *ε* = *ε*_2_ separately, then denote the corresponding potentials as $\phi_{+}^{\prime }$, $\phi_{-}^{\prime }$, and $\phi_{+}^{\prime \prime }$, $\phi_{-}^{\prime \prime }$, respectively. Thus the solution of the linearized problem, namely the potential due to two vortices, is obtained as follows: $\phi_{1}=\phi_{+}^{\prime }+\phi_{+}^{\prime \prime }$ (upper layer), $\phi_{2}=\phi_{-}^{\prime }+\phi_{-}^{\prime \prime }$ (lower layer). Finally, the interface can be derived from the linearized Bernoulli's equation (3.2):

3.3 η(x)=F2π(ρ−1){∫0+∞(P0(k0)Q′(k0)(k−k0)−P0(k)Q(k))F2ke−kTc(k;x) dk+2πP(k0)Q′(k0)Ts(k0;x)−P(k0)Q′(k0)Re[TE1]+(1−F2P0(k0)Q′(k0))(ϵ11+(x+d)2+ϵ21+(x−d)2)},

where

$$\begin{array}{rl} & Q(k)=(F^{2}k+\rho -1)\sinh(k\lambda )+\rho \gamma^{2}F^{2}k\cosh(k\lambda ),\\ & P_{0}(k)=\rho \gamma^{2}\cosh(k\lambda )+\sinh(k\lambda ),\;P(k)=F^{2}k\;e^{-k}P_{0}(k),\\ & T_{c}(k;x)=\epsilon_{1}\cos k(x+d)+\epsilon_{2}\cos k(x-d),\\ & T_{s}(k;x)=\epsilon_{1}H(x+d)\sin k(x+d)+\epsilon_{2}H(x-d)\sin k(x-d),\\ & T_{E_{1}}=\epsilon_{1}e^{ik_{0}(x+d)}E_{1}(-k_{0}+ik_{0}(x+d))+\epsilon_{2}e^{ik_{0}(x-d)}E_{1}(-k_{0}+ik_{0}(x-d)), \end{array}$$

and H( · ) means Heaviside step function, *E*_1_( · ) denotes the complex exponential integral function

$$E_{1}(z)=\int_{z}^{\infty }\frac{e^{-t}}{t}\;dt=\int_{1}^{\infty }\frac{e^{-zt}}{t}\;dt.$$

*k*_0_ is the positive root of *Q*(*k*).

Based on the stationary phase method [32], the asymptotic behaviour for the interface wave can be given by

3.4 $$\begin{array}{rl} & \eta ∼\frac{2F^{2}P(k_{0})}{\left(\rho -1)Q^{\prime }(k_{0}\right)}(\epsilon_{1}\sin k_{0}(x+d)+\epsilon_{2}\sin k_{0}(x-d))\\ & =\frac{2F^{2}P(k_{0})}{\left(\rho -1)Q^{\prime }(k_{0}\right)}\sqrt{\epsilon_{1}^{2}+\epsilon_{2}^{2}+2\epsilon_{1}\epsilon_{2}\cos(2k_{0}d)}\cdot \sin(k_{0}x+\varphi ), \end{array}$$

for large *x*, where

3.5 $$\tan\varphi =\left(\frac{\epsilon_{1}-\epsilon_{2}}{\epsilon_{1}+\epsilon_{2}}\right)\tan k_{0}d.$$

This behaviour generates from the following facts: for large *x*, H(*x* + *d*) = H(*x* − *d*) = 1. The last term, i.e. complex exponential integral functions *E*_1_( − *k*_0_ + i*k*_0_(*x* ± *d*)) and the integral in (3.3), both tend to 0 when *x* → +∞, as a simple application of Riemann–Lebesgue lemma (see [33]). The steady downstream wave height can be determined by

3.6 $$h=\frac{4F^{2}P(k_{0})}{\left(1-\rho )Q^{\prime }(k_{0}\right)}\sqrt{\epsilon_{1}^{2}+\epsilon_{2}^{2}+2\epsilon_{1}\epsilon_{2}\cos(2k_{0}d)},$$

based on this asymptotic behaviour.

For two special cases, when *ε*_1_ = −*ε*_2_

3.7 $$\begin{array}{rl} & h=\frac{4F^{2}P(k_{0})}{\left(1-\rho )Q^{\prime }(k_{0}\right)}\sqrt{\epsilon_{1}^{2}+\epsilon_{2}^{2}-2|\epsilon_{1}\epsilon_{2}|\cos(2k_{0}d)}\\ & =\frac{8F^{2}P(k_{0})}{\left(1-\rho )Q^{\prime }(k_{0}\right)}|\epsilon_{1}||\sin(k_{0}d)|; \end{array}$$

when *ε*_1_ = *ε*_2_,

3.8 $$\begin{array}{rl} & h=\frac{4F^{2}P(k_{0})}{\left(1-\rho )Q^{\prime }(k_{0}\right)}\sqrt{\epsilon_{1}^{2}+\epsilon_{2}^{2}+2\epsilon_{1}\epsilon_{2}\cos(2k_{0}d)}\\ & =\frac{8F^{2}P(k_{0})}{\left(1-\rho )Q^{\prime }(k_{0}\right)}|\epsilon_{1}||\cos(k_{0}d)|. \end{array}$$

This latter result is similar to (3.7) and the difference is only that the sine function in (3.7) is replaced by the cosine function in (3.8). In both cases, the wavelengths of the above sine-waves are both 2*π*/*k*_0_.

Moreover, the equation *Q*(*k*_0_) = 0 in terms of *k*_0_ can be rewritten as

3.9 $$\tanh(k_{0}\lambda )=\frac{\rho \gamma^{2}F^{2}k_{0}}{1-\rho -F^{2}k_{0}}.$$

Since tanh (*k*_0_*λ*) < 1, we can assert that the wavenumber *k*_0_ should be located in

3.10 $$0<k_{0}<\frac{1-\rho }{F^{2}(1+\rho \gamma^{2})}.$$

Here, we denote

3.11 $$K_{0}\;:=\frac{1-\rho }{F^{2}(1+\rho \gamma^{2})}.$$

If one takes the limit *λ* → +∞ in (3.9), then a simple result *k*_0_ = *K*_0_ is obtained. That is to say, for the upper layer with sufficient large depth, the wavenumber of the interfacial wave is approximately a fixed value *K*_0_. Besides, the analytical expression of *k*_0_ could also be derived and we put these results in appendix A.

### 3.2. Numerical method for fully nonlinear model

The numerical procedure applied in this paper is similar to that is used by [16,18–20,34]. To solve (2.6), (2.7), (2.10) and (2.13) numerically, integrals on the infinite interval are replaced by that on the truncated interval [*s*_1_, *s*_*N*_] and then calculated by the composite trapezoidal rule. Besides, half grid points are introduced to eliminate the effects of singularity in the integral. If approximate values of $y_{k}^{\prime }$, *k* = 1, 2, … on discrete grids are determined, $x_{k}^{\prime }$ could be obtained from (2.6). *x*_*k*_, *y*_*k*_ are integrated by the trapezoidal rule from the far upstream conditions (2.3). Thus the discretization of the upper-layer integral equation (2.10) leads to

3.12 $$A\phi_{1}^{\prime }=b,$$

where $\phi_{1}^{\prime }={\left[\phi_{1,2}^{\prime },\phi_{1,3}^{\prime },\ldots \right]}^{T}$. Then one gets $\phi_{1,2}^{\prime },\phi_{1,3}^{\prime },\ldots$. Next, $\phi_{2,k}^{\prime },\ldots$ on discrete grids are derived from (2.7). Calculating the discrete cost function (2.13) yields a system of equations

3.13 E(u)=0 

with respect to $u={\left(y_{2}^{\prime },y_{3}^{\prime },\ldots \right)}^{T}$, where ***E***(***u***) = (*E*_2_(***u***), *E*_3_(***u***), …). Finally, solving these equations and solutions of (2.6), (2.7), (2.10) and (2.13) are obtained. In some cases, e.g. strengths of vortices are large and the step size Δ*s* is small, numerical treatments of (3.13) lead to disastrous results. For instance, the numerical computation stagnated, or even worse, becomes divergent. This may come from the fact that the compactness of the integral operators leads to the problem of solving an ill-posed system (3.12). To overcome it, we choose the well-known Tikhonov regularization method [35]. Concretely, one can solve the normal equation

3.14 $$(A^{T}A+\alpha I)\phi_{1}^{\prime }=A^{T}b,$$

instead of (3.12), where *A*^*T*^ is the transpose of *A*, *I* is the identity matrix and *α* is the regularization parameter. If necessary, we fixed the value of *α* and adjusted it based on the performance of the numerical calculations. In our numerical experiments, an appropriate choice of the regularization parameter indeed improves the convergence speed and makes the ill-posed system become converged while the results are almost consistent with that without any regularization.

Now what matters is how to choose an efficient numerical approach to solve them. In [16,18,34], the classical Newton–Raphson method was adopted to solve the derived discrete integral equations. Each iteration step of this classical method consists of two parts: solving a Newton equation to determine the correction of the current approximate solution and then updating the approximate solution. The former needs to calculate the Jacobian matrix in each step, thus it takes up many calculating resources. Henceforth, Wang *et al.* [20] used the quasi-Newton (QN) method, a well-known class of the inexact Newton method. In each correction step, an approximate Jacobian matrix is updated by adding a matrix of rank 1 to it (see [36,37]). However, it is necessary to formulate and store an initial approximate Jacobian matrix.

The JFNK method [38], which does not need to construct the Jacobian matrix, can improve this defect especially when an efficient preconditioner could be found. While updating the approximate values of unknowns, the procedure of solving Newton equation *J*_*k*_Δ***u*** = −***E***_*k*_ is implemented by finding solutions in the Krylov space: Δ***u*** ∈ *K*_*n*_{*J*_*k*_, *r*_0_} : = span{*r*_0_, *J*_*k*_*r*_0_, …, (*J*_*k*_)^*n*−1^*r*_0_}, where *J*_*k*_ = ***E***^′^(***u***_*k*_), ***E***_*k*_ = ***E***(***u***_*k*_), *r*_0_ = −***E***_*k*_ − *J*_*k*_Δ***u***_0_ with Δ***u***_0_ the initial guess of the update (commonly set to be 0), and ***u***_*k*_ is the approximate solution in the *k*th iteration. The generalized minimal residual method (GMRES) [39], which is widely applied to solve large-scale non-symmetry linear systems, will be used in this work to implement the updating step. In the GMRES algorithm, only the evaluations of the matrix-vector product *J*_*k*_***v*** are needed and the Jacobian matrix is not constructed explicitly. This matrix-vector product can be calculated by the forward difference

$$\frac{E(u_{k}+\varepsilon v)-E(u_{k})}{\varepsilon }\approx J_{k}v,$$

where ɛ > 0 is small sufficiently.

In practice, an efficient preconditioner will improve the convergence of the GMRES program and decrease the time consumption significantly. Here, we choose the right preconditioning method, thus the solving process of the Newton equation is split into two steps: firstly solving *J*_*k*_*P*^−1^*z* = −***E***_*k*_, *z* ∈ *K*_*n*_{*J*_*k*_*P*^−1^, *r*_0_}, then we obtain Δ***u*** = *P*^−1^ *z* and ***u***_*k*+1_ = ***u***_*k*_ + Δ***u***, where *P* is the right preconditioner. Motivated by the works of [40,41], in the following, we construct a preconditioner via the following procedure: building corresponding boundary integral equations (BIEs) based on the linearized problems, then calculating the Jacobian matrix of the discrete version of BIEs and storing it as the preconditioner.

By virtue of the linearized problem in §3.1, we establish the BIEs for the linearized problem again along both upper and lower layers. The new integral path for the upper layer consists of *y* = 0, *y* = 2*λ*, a semicircle that centres *z* = *z*(*s*) and bypasses it in the upper layer, and two vertical lines *x* = ±*L*, *L* → +∞. Meanwhile, the new integral path for the lower layer consists of *y* = 0, a semicircle centring *z* = *z*(*s*) and bypassing it in the lower layer, and a semicircle immersed in the lower layer with an infinite radius. Next, for the sake of the convenience of presentations, we introduce the following denotements:

$$U_{1}(x)\;:=\frac{\partial \phi_{1}}{\partial x}(x,0),\;U_{2}(x)\;:=\frac{\partial \phi_{2}}{\partial x}(x,0),\;V(x)\;:=\frac{\partial \phi_{2}}{\partial y}(x,0)=\frac{1}{\gamma }\frac{\partial \phi_{1}}{\partial y}(x,0),$$

and use the BIEM in §2 associated with linearized boundary conditions (3.1), (3.2), the kinematic condition on the upper surface of the upper layer (2.5), then we have the following two BIEs:

3.15 $$\pi \left(\gamma -U_{1}(x)\right)=\int_{-\infty }^{+\infty }\frac{\gamma V(t)}{t-x}\;dt+\int_{-\infty }^{+\infty }\frac{\left|\begin{array}{cc} t-x & -2\lambda \\ \gamma -U_{1}(t) & \gamma V(t) \end{array}\right|}{{\left(t-x\right)}^{2}+4\lambda^{2}}\;dt$$

and

3.16 $$\pi \left(U_{2}(x)-1\right)=\int_{-\infty }^{+\infty }\frac{V(t)}{t-x}\;dt+\frac{\epsilon_{1}}{{\left(x+d\right)}^{2}+1}+\frac{\epsilon_{2}}{{\left(x-d\right)}^{2}+1}.$$

(3.1) and (3.2) become

3.17 $$\rho \gamma U_{1}(x)-U_{2}(x)+\frac{\rho -1}{F^{2}}\eta (x)=0,$$

with a by-product

3.18 $$\eta (x)=\int_{-\infty }^{x}V(t)\;dt.$$

Taking (3.16) as the cost function ***E***(*x*), then the discrete version of the system (3.15), (3.16), (3.17), (3.18) are denoted by (*E*_*j*_) with respect to *V*_*j*_, *j* = 1, 2, …, where *E*_*j*_ : = *E*(*x*_*j*_), *V*_*j*_ : = *V*(*x*_*j*_) and *x*_*j*_ are the discrete grids. Finally, we can derive its Jacobian matrix as the preconditioner *P* = (*P*_*ij*_), where *P*_*ij*_ : = ∂*E*_*i*_/∂*V*_*j*_. Besides, for saving stores the GMRES(m) algorithm, namely restarting the GMRES algorithm after m steps, is used in practical computations.

## 4. Results analysis

In this section, we will discuss the linear and nonlinear internal wave profiles due to two point vortices using the linearized theory and fully nonlinear boundary integral equation model proposed in §3. Comparison between linear and nonlinear results is given to show the obvious nonlinear characteristics and to illustrate the difference from linear results. We will give two cases, counter-rotating point vortices and co-rotating point vortices, which include the wave profiles, amplitudes of steady downstream waves, amplitudes of first waves, and the effect of vortex strength and distance between two vortices. Some detailed parameters are given as follows. The depth of the upper layer is set *λ* = 20, and far upstream uniform speed *γ* = 1. If *ε*_1_ = 0 or *ε*_2_ = 0, this case degenerates to the case for a single vortex. The symbol *h* represents the downstream wave height due to the vortex pair, which is the distance of two adjoint wave crest and wave trough of steady downstream waves, and *h*_1_, *h*_2_ represent wave heights due to one single vortex *ε*_1_, *ε*_2_ separately. If necessary, the symbol *ε* represents the strength of one vortex when comparing the results of a vortex pair and only one vortex. The Froude number *F* = 0.13, and the density ratio *ρ* is set to be 0.9. Hence from (3.9) the linear downstream wavenumber *k*_0_ = 3.114 and the corresponding linear wavelength is

4.1 $$L_{0}=\frac{2\pi }{k_{0}}=2.018.$$

For other combinations of parameters *F*, *ρ*, similar phenomena could also be observed. Besides, if *ρ* is small (for example, *ρ* < 0.8), the interfacial waves are significantly small. Thus only the case of large *ρ* (e.g. *ρ* ≥ 0.9) is worth concern. In most cases, the calculation domain is [−25, 30], the grid number *N* = 2201, and the step size Δ*s* = 0.025. The tolerance error is *σ* = 10^−9^.

**Figure 2.**
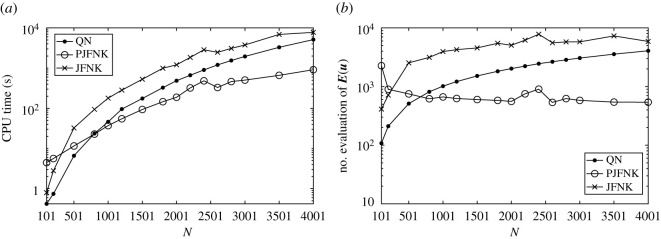
Comparisons of performances of different methods: Quasi-Newton (QN) method, Jacobian-free Newton–Krylov (JFNK) method without the preconditioner, and preconditioned Jacobian-free Newton–Krylov (PJFNK) method with different grid numbers *N*. (*a*) The CPU time(s). (*b*) The number of evaluations of the nonlinear function ***E***(***u***).

Following is the comparison of performance among the QN method used in [20], the JFNK method without any preconditioner and the preconditioned Jacobian-free Newton–Krylov (PJFNK) method in this work. The calculation domain is fixed as [−25, 30]. Figure 2 shows the comparisons of CPU times and the number of evaluations of the nonlinear function ***E***(***u***), which comes from equation (3.13), with various values of the grid number *N*. Vortex strengths are set to be *ε*_1_ = −0.24, *ε*_2_ = +0.24, and the distance between two vortices is *d* = 0.51. Other parameters are the same as above. The number of the maximum dimension of the Krylov space *m* is set to be 20 for the PJFNK method and 100 for the JFNK method without a preconditioner to ensure the convergence. The regularization parameter *α* is applied when *N* ≥ 2601. This causes an abrupt decrease near *N* = 2501 in figure 2.

From figure 2, the JFNK with the preconditioner built in the previous section is more efficient than the rank-1 Boryden QN method used in [20] when *N* is large. A naive application of the JFNK method without any preconditioner possesses the worst performance. Besides, the PJFNK makes the number of evaluations of ***E***(***u***) around several hundred even *N* = *O*(10^3^), while it increases fast with *N* for the other two methods. This may be the main reason why the PJFNK method has the best performance.

### 4.1. Counter-rotating vortices

In this subsection, we consider the nonlinear effects of two point vortices and their interactions. we compare the linear results provided in §3.1 and nonlinear results provided in §3.2 of two vortices with different distances and strengths. Especially, when the strengths of vortices are large, the regular parameter, as we have described in §3.2, may be necessary. A typical choice of this regular parameter is *α* = 0.02.

#### 4.1.1. Effects of exchanging signs of the vortex pair

**Figure 3.**
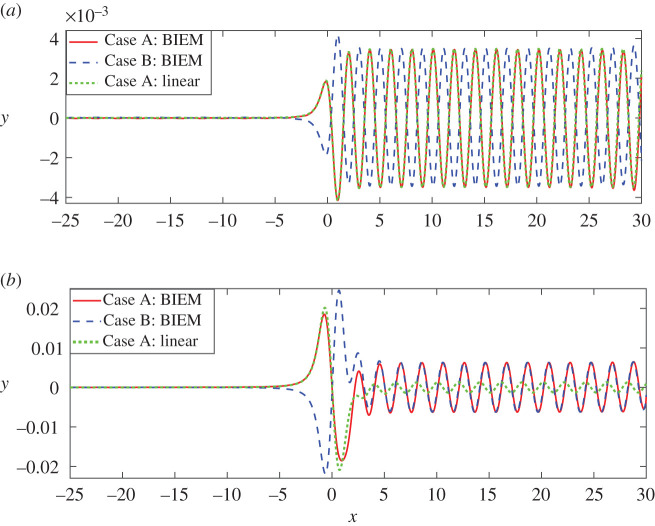
Interface wave profiles when exchanging signs of counter-rotating pairs for different *d*. Other parameters *F* = 0.13, *ρ* = 0.9. Case A corresponds to *ε*_1_ = −0.24, *ε*_2_ = +0.24, and Case B corresponds to *ε*_1_ = +0.24, *ε*_2_ = −0.24. (*a*) *d* = 0.05 and (*b*) *d* = 0.99.

For convenience, we use the following denotements. Case A: *ε*_1_ = −0.24, *ε*_2_ = +0.24; Case B: *ε*_1_ = +0.24, *ε*_2_ = −0.24. For linear solution (3.3), one can see that changing the signs of two vortices simultaneously leads to the following results: the interface of the linear solution *η*(*x*) becomes −*η*(*x*), i.e. its image about the still interface *y* = 0. This suggests that linear solutions for Case A are symmetric about *y* = 0 with that for Case B. Whereas this may not happen for nonlinear solutions. Here, we have plotted two typical comparisons in figure 3 for Case A and Case B, where the distances between the vortex pair are chosen deliberately. Firstly, figure 3*a* is for a very small distance *d* = 0.05 and figure 3*b* is for a larger distance *d* = 0.99. In figure 3*a*, the symmetric relationships between the wave profiles for both Case A and Case B are identified for nonlinear results. Besides, for Case A both linear and nonlinear results coincide, thus one may deduce that the nonlinear effect is weak at this time. By the way, in this time the wave profiles are characterized by the similar amplitudes between the first two waves and the downstream waves, which occurs in the case of a dipole. This suggests that when the distance between two vortices is small, the wave profiles may be approximately considered as that due to a dipole. Next, in figure 3*b*, in contrast to figure 3*a*, the first wave of Case B is larger than that of Case A. Meanwhile, the amplitudes of the first waves are larger than that of the steady downstream wave. Although the first waves of Case A and Case B are not strictly symmetric, the nonlinear steady downstream wave heights coincide with each other very well and both of them are much bigger than the linear wave heights in the area of downstream waves. Given the above, the vortex pair with a big distance shows strong nonlinear effects in figure 3*b*.

#### 4.1.2. Effects of the distance of the vortex pair

**Figure 4.**
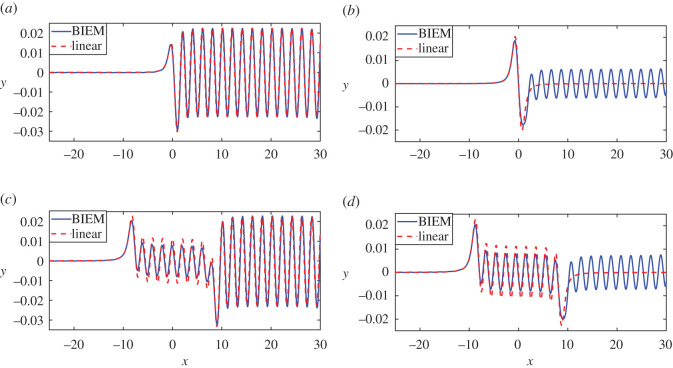
Comparison between nonlinear and linear solution for different *d*. Other parameters *F* = 0.13, *ρ* = 0.9 and *ε*_1_ = −0.24, *ε*_2_ = 0.24. (*a*) *d* = 0.51, (*b*) *d* = 1.01, (*c*) *d* = 8.575 and (*d*) *d* = 9.079.

From the above, we can assume that distance between two vortices is a characteristic factor that influences the wave profiles. Next, we focus on the discussion of the influences of the distance between two vortices. From the above discussions, we know that downstream wave heights of both Case A and Case B are almost the same. Besides, from numerical experiments, we have confirmed that this point does not change no matter how *d* varies. Thus we only focus on Case A in this subsection. Both wave profiles of linear and nonlinear solutions are shown in figure 4 for the vortex pair in Case A, while four sub-figures are corresponding to different distances between two vortices. Specifically, in these sub-figures, the distance between two vortices 2*d* is close to (*a*) 0.5*L*_0_, (*b*) 1.0*L*_0_, (*c*) 8.5*L*_0_, (*d*) 9.0*L*_0_, where *L*_0_ from (4.1) is the linear downstream wavelength. The first crest and trough are similar to the amplitude of downstream waves in both sub-figures 3*a* (*d* = 0.05) and 4*a* (*d* = 0.51). But when *d* becomes bigger gradually, the first crest and trough are quite uplifted and larger than the amplitude of downstream waves in figure 4*b*. This fact could be explained as the produced waves weaken each other and mainly show features of wave profiles due to a dipole if two vortices are near, such as shown in figures 3*a* and 4*a*.

The first wave caused by a single negative vortex *ε* < 0 is a crest, while the first wave caused by a single positive vortex *ε* > 0 is a trough. This fact is not only implied by the expression of linear results but also provided by fully nonlinear results as shown in [18]. In figure 4, the first crest also corresponds to the negative vortex and the first trough is related to the positive one.

We observe the phenomenon that wave profiles for nonlinear and linear solutions are almost the same in figures 3*a* and 4*a* when *d* is small. But wave profiles, especially steady downstream wave profiles of nonlinear and linear solutions after the second vortex are different for a larger distance. Figure 4*b*–*d* shows the wave profiles with a large distance which is about one to several times downstream wavelengths. On the one hand, from (3.7), when *d* is set to make 2*d* = *mL*_0_, m∈N, where 2*d* is the distance between two vortices and *L*_0_ is the linear downstream wavelength, the linear downstream wave profile is just like a horizontal line since the linear solution satisfies superposition law and the waves can weaken each other, as shown in figure 4*b*,*d*. Meanwhile, the nonlinear downstream wave is obviously different from linear results because the nonlinear solution does not satisfy the superposition principle and the nonlinear effect on downstream wave profiles is strong. Similarly, in figure 4*d* with *d* = 9.079, the linear wave profile is flat, owing to the elimination of both point vortices, and the nonlinear results still give obvious wave profiles. By the way, as the distance between the vortex pair is large, waves caused by the first vortex are not influenced by the second one, thus one can find the obvious wave profiles before the second vortex in figure 4*d*. On the other hand, the steady downstream waves in figure 4*c* with *d* = 8.575 (about 8.5*L*_0_) enlarge and coincide very well between linear and nonlinear results. As described, these results suggest destructive and constructive interference occur, owing to the interactions between waves due to the upstream and downstream vortex separately. Besides the downstream wave profiles after the second vortex, we also note that if the distance between two vortices is large enough the waves between two vortices are almost fixed and are similar to wave profiles due to one single vortex *ε* = *ε*_1_, while the downstream waves vary with the increase of *d* since the wave interference. The linear results and nonlinear results verified each other well before the second vortex.

**Figure 5.**
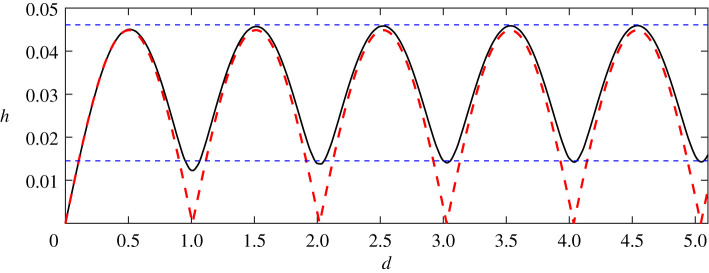
Relationship of downstream wave heights and distances of two vortices for the nonlinear and linear solution, where the black solid curve represents the wave height of the nonlinear solutions, the red dashed curve depicts that of the linearized solutions obtained in (3.7). Two horizontal lines (dashed) represent *h*_+_ ± *h*_−_, where *h*_+_ = 0.0303, *h*_−_ = 0.0158 are the nonlinear wave height for a single vortex *ε* = −0.24 and *ε* = +0.24, respectively.

In the following, we analyse how wave height of steady downstream waves for nonlinear and linear solutions change with the distance of two point vortices increasing in figure 5. The resolution in figure 5 for nonlinear results (black solid curve) is Δ*d* = 0.05, and a finer resolution Δ*d* = 0.01 is used if the local resolution refinement is needed. The dashed line denotes the linear solution, and the solid line denotes nonlinear results. We have given the analytical expressions (3.7) when *ε*_1_ = −*ε*_2_, from which one can claim that the linear downstream wave height changes periodically with the distance between two vortices in the form of a sine function and the period is *π*/*k*_0_ = *L*_0_/2. The maximum values of downstream wave heights for a counter-rotating pair are doubled for the single point vortex and the minimum values are zero for linear results since linear solutions obey the linear superposition principle. This can be obtained while setting *ε*_1_ = *ε*, *ε*_2_ = −*ε* and *ε*_1_ = *ε*, *ε*_2_ = 0 in (3.6), then comparing the results.

Especially, both linear and nonlinear results change periodically and show the same shapes in figure 5. In addition, the maximum values of the linear and nonlinear results are corresponding to the same distance, and so are the minimum values. However, the wave height of steady downstream waves for nonlinear results is always bigger than that for linear results with any distance between two counter-rotating point vortices. The linearized solution (3.7) predicts zero downstream waves whenever the wavenumber *k*_0_ has values obtained from

$$k_{0}d=n\pi ,\;n=1,2,3,\ldots ,$$

but the nonlinear results do not behave this way. The minimum for nonlinear results is quite larger than that for linear results. For the nonlinear results, because the amplitudes of steady downstream waves are different for negative and positive vortices, even for the same vortex strength the minimum values, which are results of the destructive interference between waves due to the first and the second vortex separately, do not vanish and the maximum value, 0.0459, is about 3.7 times larger than the minimum value, 0.123, for nonlinear results, as shown in figure 5. In this subsection, we use the grid number *N* = 2201 and the step size is 0.025, which is consistent with that in most cases. Since it does not significantly improve the precision of the nonlinear results when *N* ≥ 2201 even in the case where *h* reaches the minimum, we do not use a larger grid number. However, if a possible waveless solution is found, it is wise to increase *N*, and the convergence can be retained if an appreciated regularization strategy is applied (e.g. a better method of determining the regularization parameter), as shown in the previous numerical experiments in figure 2.

In addition to the effects on wave height, different values of *d* have significant influences on the phase of Case A and Case B. When *d* is small (at this time the setting of two equal-strength contour-rotating vortices can be seen as that of a dipole) or is set to be specific values that make the downstream wave heights reach the minimum as discussed above, downstream wave profiles of Case A and Case B have opposite phase, while *d* is set to be specific values related to the maximum of the wave heights, the phases of Case A and Case B coincide. For other values of *d*, there still exist phase differences but constructive/destructive interference does not happen. This may be explained as follows: on the one hand, wave profiles due to the first vortex (referred to as ‘wave 1’) and the second vortex (referred to as ‘wave 2’) separately have almost the same phase in the former situation, then constructive interference occurs and the phase depends on the sign of the vortices. Specifically, the phase of wave 1/wave 2 determines the phase of the downstream waves and we only consider wave 1 as the determining factor. From [18], we have known that wave 1 has opposite phase if one changes the sign of the first vortex. Besides, from (3.4) and (4.1), the downstream wavelength is *L*_0_. This is still suitable for wave 1. From (3.7) and the discussion above, the period of downstream wave height *h* with respect to *d* is *L*_0_/2 and the maximum points are *d* = *L*_0_/4 + *m*(*L*_0_/2), m∈N. These can also be applied for nonlinear results. At this time, wave 1 moves upstream with a quarter of wavelength, and changing the sign of the first vortex leads to the opposite phase of wave 1. So the opposite phase of downstream waves for Case A and Case B occurs due to the opposite phase of wave 1. On the other hand, in the case that *h* reaches its minimum value, *d* equals *L*_0_/2 + *m*(*L*_0_/2), m∈N. Meanwhile, wave 1 and wave 2 have opposite phases and destructive interference occurs. However, from [18], wave 1 and wave 2 have different wave heights since their signs are opposite. Thus the phase is determined by the wave profiles for which wave height is larger. If the height of wave 1 is larger, then one can see that wave 1 moves upstream with a half wavelength; if that of wave 2 is larger, then one can see that wave 2 moves downstream with a half wavelength. Both cases lead to the fact that the phase of the wave profiles which wave height is larger are consistent for both Case A and Case B. Therefore, phases of downstream waves for both Case A and Case B are the same in the latter situation.

#### 4.1.3. Effects of the strengths of the vortex pair

**Figure 6.**
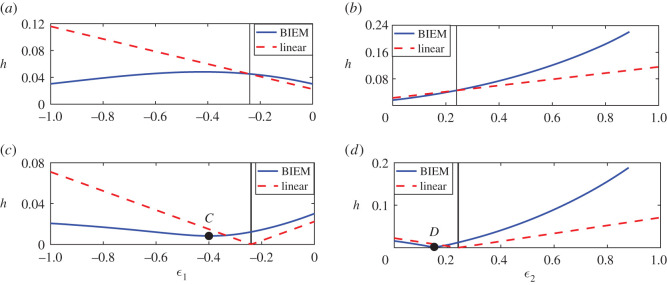
Comparison of wave height for nonlinear and linear solutions when fixing one vortex and changing another. Two minimum points are: *C*(−0.4, 0.008), *D*(0.15, 0.002). The vertical line represents the case of a symmetric counter-rotating pair *ε*_1_ = −0.24, *ε*_1_ = 0.24. (*a*) The height of downstream waves at the maximum point in figure 5 for different first vortex strength *ε*_1_ with *d* = 0.51 and *ε*_2_ = 0.24. (*b*) The height of downstream waves at the maximum point in figure 5 for different second vortex strength *ε*_2_ with *d* = 0.51 and *ε*_1_ = −0.24. (*c*) The height of downstream waves at the minimum point in figure 5 for different first vortex strength *ε*_1_ with *d* = 1.01 and *ε*_2_ = 0.24. (*d*) The height of downstream waves at the minimum point in figure 5 for different second vortex strength *ε*_2_ with *d* = 1.01 and *ε*_1_ = −0.24.

To further investigate the effects of interference, in figure 6, we examine the comparisons between fully nonlinear BIEM results and linear results at maximum and minimum points *d* = 0.51 (about *L*_0_/4) and 1.01 (about *L*_0_/2) in Case A. We fix the strength of one vortex and change another to compare the wave heights of steady downstream waves of nonlinear and linear results. Especially, figure 6*a*,*c* (figure 6*b*,*d*) shows the wave height of steady downstream waves with respect to the strength of the first negative vortex *ε*_1_ (the second vortex *ε*_2_) while the second positive vortex remains *ε*_2_ = 0.24 (the first vortex strength remains *ε*_1_ = −0.24). The dashed line denotes linear results and the solid line denotes fully nonlinear results. Figure 6*a*,*b* corresponds to the first maximum point *d* = 0.51 in figure 5, at the same time figure 6*c*,*d* corresponds to the minimum point *d* = 1.01 in figure 5. Hence, from (3.6) and (4.1) one can obtain the expressions of linear downstream wave height

4.2 $$h=\frac{4F^{2}P(k_{0})}{\left(1-\rho )Q^{\prime }(k_{0}\right)}|\epsilon_{1}-\epsilon_{2}|,$$

for *d* = 0.51 and

4.3 $$h=\frac{4F^{2}P(k_{0})}{\left(1-\rho )Q^{\prime }(k_{0}\right)}|\epsilon_{1}+\epsilon_{2}|,$$

for *d* = 1.01, respectively. Thus, for the former case, the linear wave height increases proportionally with *ε*_1_(*ε*_2_) increasing. For the latter case, it decreases to zero and then increases as *ε*_1_(*ε*_2_) goes up from 0. These correspond to the dashed lines in sub-figures 6*a*,*b* and polylines in sub-figures 6*c*,*d*. Furthermore, for nonlinear results, the downstream wave height also increases when *ε*_1_(*ε*_2_) becomes stronger, but the increasing rates are characterized by the sign of the vortex: in figure 6*a* the nonlinear wave height increases slower than the linear one and then gradually decreases, but in figure 6*b* it goes up faster than the linear one significantly. Besides, increasing the strength of the positive vortex leads to the nonlinear wave height surpassing the linear counterpart. Typically, when the distance between two vortices 2*d* is close to *L*_0_, i.e. *d* = 1.01, the destruction interference occurs. From [18], we have known that increasing the strength of one vortex leads to both the increase of nonlinear downstream wave height and the movement of locations of crests/troughs of the nonlinear downstream wave profiles. Besides, for fully linear solutions the downstream wave height due to a positive vortex is larger than that due to an equal-strength negative counterpart. These features make a consequence that the minimum point of the nonlinear wave height, namely the point where the downstream wave profiles are weakened as much as possible, does not correspond to the case of equal-strength pair and the minimum is not 0, and violates the predictions of the linear solution. Especially, they are about 0.0083 with respect to *ε*_1_ = −0.4 (the point *C*) in figure 6*c* and 0.0018 with respect to *ε*_2_ = 0.15 (the point *D*) in figure 6*d*.

On the other hand, in figure 6*a*,*b*, the nonlinear and linear results have the intersection point at *ε*_1_ = −0.24, *ε*_2_ = 0.24 while *d* = 0.51, which represents the first maximum point in figure 5. At this point, the nonlinear effects on the interface are strongly weakened and the wave profiles of nonlinear and linear results are close like figure 4*b* shows. In figure 6*c*,*d,* the intersection points are at *ε*_1_ = −0.335, *ε*_2_ = 0.24 and *ε*_1_ = −0.24, *ε*_2_ = 0.187 separately when *d* = 1.01, which represents the first minimum point in figure 5. At both points, the downstream wave heights for nonlinear and linear results are the same but the wave profiles may be different.

**Figure 7.**
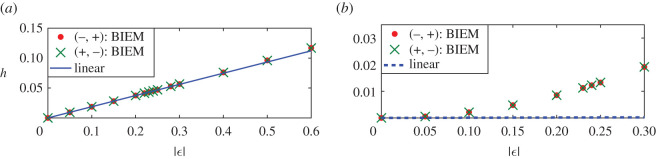
Wave heights of nonlinear and linear solutions due to counter-rotating pairs for different vortex strength. In sub-figure (*b*), the wave height of the linear solution (dashed line) is always 0. ( − , + ) means *ε*_1_ < 0, *ε*_2_ > 0, and ( + , − ) meant *ε*_1_ > 0, *ε*_2_ < 0. Linear results are independent of signs of the vortex pair. (*a*) *d* = 0.51 and (*b*) *d* = 1.01.

**Figure 8.**
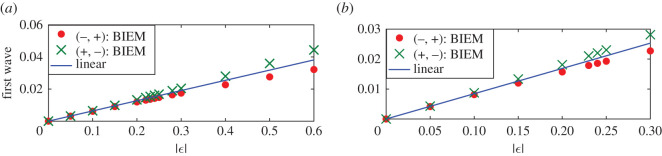
Amplitudes of the first wave for nonlinear and linear solutions due to counter-rotating pairs for different vortex strength. (−, +) means *ε*_1_ < 0, *ε*_2_ > 0, and (+, −) meant *ε*_1_ > 0, *ε*_2_ < 0. Linear results are independent of signs of the vortex pair. (*a*) *d* = 0.51 and (*b*) *d* = 1.01.

If the two point vortex strengths increase equally with opposite signs, figures 7 and 8 give the steady downstream wave height and the amplitudes of the first wave with respect to the vortex strength. Figures 7*a* and 8*a* are corresponding to the first maximum point *d* = 0.51; in other words, at this time the distance between two vortices is about *L*_0_/2 and the constructive interference of downstream waves due to two separate vortices occur. And figures 7*b* and 8*b* are corresponding to the first minimum point *d* = 1.01 in figure 5 or the case that the destructive interferences happen. For both cases, the wave heights of nonlinear steady downstream wave are the same for both cases *ε*_1_ < 0, *ε*_2_ > 0 and *ε*_1_ > 0, *ε*_2_ < 0 as shown in figure 7*a*,*b*. Besides, nonlinear and linear results coincide with each other very well no matter how the vortex strengths increase in figure 7*a*, while the nonlinear wave heights are not weakened to 0 like linear results and still increase with the vortex strengths. Instead, figure 8 shows that amplitudes of the first wave for case *ε*_1_ > 0, *ε*_2_ < 0 are larger than that for linear results, and the latter is larger than amplitudes of the first wave for case *ε*_1_ < 0, *ε*_2_ > 0. The difference between nonlinear results for cases *ε*_1_ < 0, *ε*_2_ > 0 and linear results is slightly larger than that between nonlinear results for cases *ε*_1_ > 0, *ε*_2_ < 0 and linear results. Both of them increase slowly with vortex strength increasing, as figure 8*a*,*b* demonstrates. Except for the case that the distance between two vortices is about multiple times *L*_0_, the linear solution can predict the downstream wave height and the amplitude of the first wave due to an equal-strength counter-rotating pair even the vortex strength is large, which may be a consequence of the neutralization between effects of a negative vortex and a positive one.

### 4.2. Co-rotating vortices

Internal wave induced by co-rotating vortex pair is discussed in this subsection, including the wave profiles, steady downstream wave affected by the distance between two vortices as well as vortex strength, the first wave affected by vortex strength. Constructive and destructive interference also occurs. Besides, we observe the shapes of trapped waves clearly under special cases.

#### 4.2.1. Effects of the distance of the vortex pair

**Figure 9.**
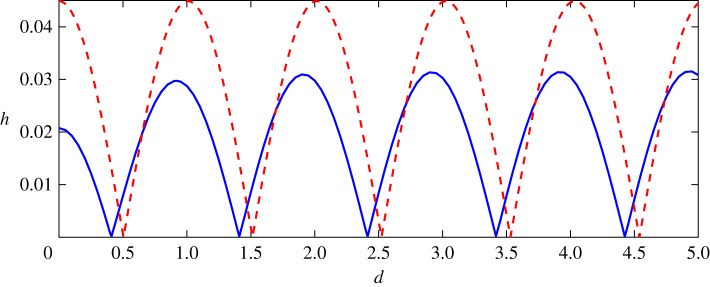
Relationship of wave height of downstream wave profiles and distance between two vortices for the nonlinear (solid) and linear (dashed) solution for *ε*_1_ = *ε*_2_ = −0.24.

**Figure 10.**
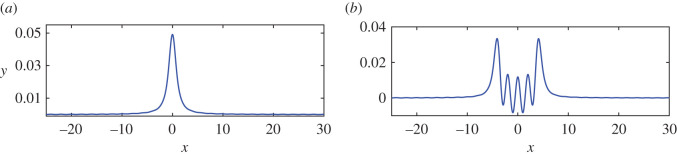
(*a*) Nonlinear wave profiles when *ε*_1_ = *ε*_2_ = −0.42 and *d* = 0.319. (*b*) Typical wave profiles that are ‘trapped’ between two vortices when *ε*_1_ = *ε*_2_ = −0.42 and *d* = 4.334. (*a*) Single wave, (*b*) trapped wave.

Figure 9 shows the periodical relation between the amplitude of steady downstream waves and the distance of the negative vortex pair *ε*_1_ = *ε*_2_ = −0.24. Both nonlinear results denoted by the solid line and linear results denoted by the dashed line are periodical with respect to the distance of the vortex pair. They have nearly the same period and the nonlinear result reaches minimum and maximum points advance since the curve for nonlinear solutions moves left about 0.1. This comes from the fact that the locations of crests/troughs of nonlinear wave profiles due to one negative vortex move upstream as its strength increases. Besides, the maximum amplitude of nonlinear results is only about 3/4 of that of linear results for *d* > 1, and low to about 1/2 for a much smaller *d*. Since both vortices have the same signs, they would induce bigger waves according to linear theory. However, the nonlinear results for distance 0 < *d* < 0.469, one of the intersection points between solid and dashed line in figure 9, gives smaller amplitudes than either linear results or nonlinear results for *d* > 0.5. Finally, in contrast with the case of contour-rotating pairs, the downstream waves can be almost weakened to be flat, and special 'waveless' solutions can be found. For instance, figure 10 shows two cases: (*a*) the whole wave profile contains only one wave; (*b*) waves are 'trapped' between two vortices, namely, the trapped wave occurs.

**Figure 11.**
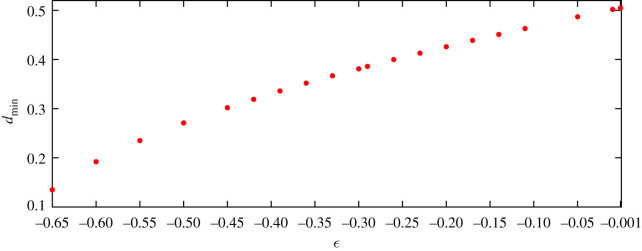
Relationships of *d*_min_ and vortex strength of two vortices for nonlinear results. If the distance between two vortices is 2*d*_min_ and the vortex strengths are *ε*_1_ = *ε*_2_ = *ε* < 0, the downstream wave heights are almost zero and the whole wave profile consists of only one wave. The value of *d*_min_ for linear results is still *π*/*k*_0_ = 0.504 from (3.6) no matter how large the vortex strength is.

To explore the possible occurrence conditions of the case of one single wave like figure 10*a* depicts, we denote *d*_min_ as the smallest value of *d* that makes the steady downstream wave height become 0. For instance, in figure 9, when *ε*_2_ = *ε*_2_ = −0.24, we have *d*_min_ = 0.410. Figure 11 shows that this minimum point *d*_min_ decreases as *ε* strengthens from 0. The curve in figure 11 can be fitted by *d*_min_ = 0.8197*ε*^3^ + 0.3521*ε*^2^ + 0.448*ε* + 0.5074, namely, the rate of change of *d*_min_ with *ε* is not constant, so the decrease is not linear. When *ε* = 0.001, *d*_min_ is 0.505, which is close to 0.504, (*L*_0_/4), the first minimum point of linear results in figure 9. Conversely, if the distance of two vortices 2*d* is *L*_0_/2, for linear solution the wave profiles always consist of only one wave no matter how the strengths of two vortices change.

**Figure 12.**
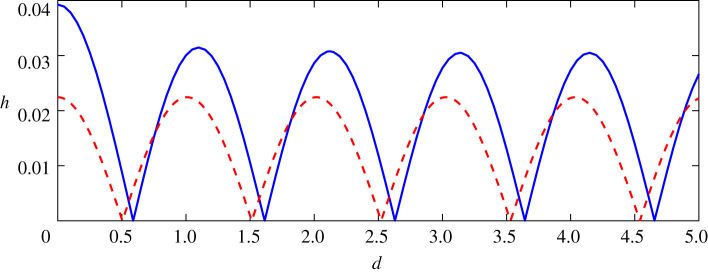
Relationship of wave height of downstream wave profiles and distance between two vortices for the nonlinear (solid) and linear (dashed) solution for *ε*_1_ = *ε*_2_ = +0.24.

As for two equal-strength positive pairs, figure 12 shows the wave heights of steady downstream waves with respect to the distance between two positive vortices with *ε*_1_ = *ε*_2_ = 0.24. Similar to the case of both negative point vortices as shown in figure 9, linear and nonlinear results are periodical along with the distance between two vortices and both of them can reach zero, which means that there are no waves behind the second vortices for some suitable distance of two vortices, namely, the waves are trapped between two vortices. Furthermore, the zero points of nonlinear results are slightly bigger than that of linear results. The maximum amplitudes of nonlinear results are bigger than that of linear results, especially for the small distance between the vortex pair. The nonlinear interaction enhanced the amplitudes of steady downstream waves, which is bigger than the linear superposition of two point vortices’ amplitudes.

Compared with the results of the counter-rotating vortex pair as shown in figure 5, the difference between nonlinear results and linear results of co-rotating vortex pairs is obvious in figures 9 and 12. Firstly, for counter-rotating pairs, both linear and nonlinear results have the same locations of maximum/minimum, while the difference of locations of these extreme points between linear and nonlinear is determined by the signs of two vortices. Secondly, for co-rotating pairs, both minimum amplitudes of linear and nonlinear results can reach zero, but only linear results could reach zero for counter-rotating vortex pairs. Thirdly, when the distance of the vortex pair is small, there are obvious distinctions between linear and nonlinear amplitudes for co-rotating vortex pairs. Whereas the wave profiles of both nonlinear and linear results near coincide when the distance of the vortex pair is small.

#### 4.2.2. Effects of the strengths of the vortex pair

**Figure 13.**
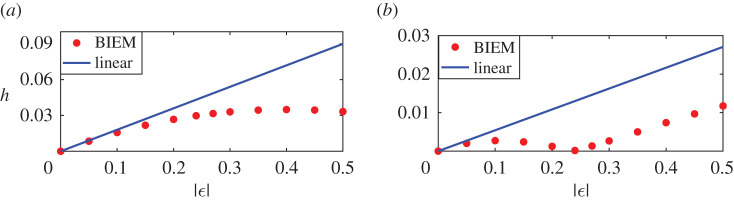
Steady downstream wave heights for nonlinear and linear solutions due to co-rotating pairs *ε*_1_ = *ε*_2_ < 0 for different vortex strength. (*a*) *d* = 0.919 and (*b*) *d* = 0.410.

**Figure 14.**
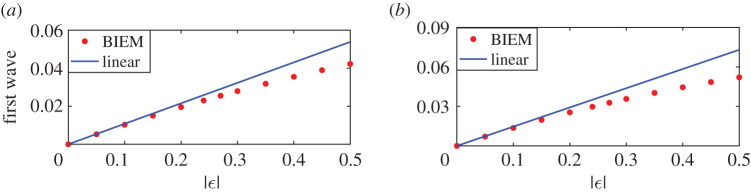
Amplitudes of the first wave for nonlinear and linear solutions due to co-rotating pairs *ε*_1_ = *ε*_2_ < 0 for different vortex strength. (*a*) *d* = 0.919 and (*b*) *d* = 0.410.

Figure 13 shows the effects of different strengths on linear and nonlinear steady downstream waves for negative co-rotating vortex pairs. Figure 13*a*,*b* is corresponding to the distance between vortex pair *d* = 0.410 and *d* = 0.919, namely, the first minimum/maximum point in figure 9. Noting that now we do not consider the case that the distance of two vortices is *L*_0_/2 or *L*_0_, thus the linear wave height is neither sum of that due to two vortex separately nor 0. The linear amplitudes increase linearly with the increase of vortex strength, which could also be deduced from the analytical expression (3.3), and the nonlinear amplitude is more complicated. When *d* = 0.410, the wave height of nonlinear results increases first slowly from zero, then decreases to zero, and continues to increase with the increase of the strength of the vortex pair as shown in figure 13*b*, but is still lower than the linear counterpart. On the other, when *d* = 0.919, the wave height of nonlinear steady downstream waves increases continuously and then decreases as |*ε*| > 0.3, but the difference to linear results is bigger and bigger with the increasing strength of the vortex pair. Next, figure 14 shows the amplitudes of the first wave for the negative vortex pair. Both linear and nonlinear amplitudes increase monotonously along with increasing strength of vortex pair for either *d* = 0.410 or *d* = 0.919. As discussed above, both amplitudes and the downstream wave heights are less than their linear counterparts.

**Figure 15.**
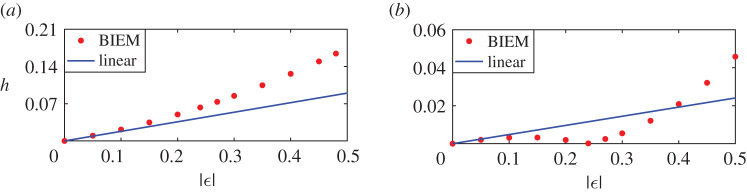
Steady downstream wave heights for nonlinear and linear solutions due to co-rotating pairs *ε*_1_ = *ε*_2_ > 0 for different vortex strength. (*a*) *d* = 1.098 and (*b*) *d* = 0.588.

**Figure 16.**
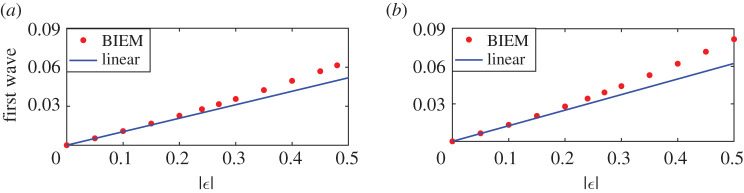
Amplitudes of the first wave for nonlinear and linear solutions due to co-rotating pairs *ε*_1_ = *ε*_2_ > 0 for different vortex strength. (*a*) *d* = 1.098 and (*b*) *d* = 0.588.

Moreover, figures 15 and 16 show the wave heights of steady downstream waves and the amplitudes of the first waves induced by the positive vortex pair with respect to the strength of the vortex pair. The sub-figures 15*a*, 16*a* and 15*b*, 16*b* correspond to the distance of vortex pair *d* = 1.098 and *d* = 0.588, respectively. Compared with figures 13 and 14, curves in figures 15 and 16 have similar shapes, but the nonlinear results are still larger than the linear counterparts. Even in figure 15*b*, the nonlinear wave height firstly goes to zero and then increases much faster and goes over the linear results. Besides we can easily find the first trough is bigger than amplitudes of steady downstream waves both for nonlinear and linear results when *d* = 0.588. This phenomenon illustrates that the distance between the vortex pair could change the amplitudes of both steady downstream waves and the first trough greatly. This has been found to be true both for the nonlinear solution, as well as for the linearized approximation.

## 5. Conclusion

Interfacial waves due to a vortex pair submerged in steady two-dimensional two-layer ideal fluids flow are studied and a related fully nonlinear boundary integral equation model is established. We have introduced a matrix form in the boundary integral equations, thus the expressions are more compact and easier to write. Linear analytical solutions of the wave profile induced by a vortex pair are given and the asymptotic behaviour for steady downstream waves is provided. The JFNK method is applied to solve the fully nonlinear model. Wave profiles, steady downstream wave heights, and amplitudes of the first wave of both nonlinear and linear results for counter- and co-rotating pairs are discussed.

In the case of equal-strength counter-rotating pairs, we consider the relationship between downstream wave height and the distance of two vortices, in the sense of linear and nonlinear methods. They all change periodically with consistent locations of maximum and minimum points, and they take on the same trend with distance. The maximum values of linear and nonlinear results correspond to the same distance, and the same is true of the minimum values. According to (3.6), when *d* = *mπL*_0_/2 (*m* even), the downstream wave height of the linear solution is zero, while the minimum value of the downstream wave height of the nonlinear solution is not zero. Therefore, it is necessary to consider nonlinear effects. Fixing the first vortex strength *ε*_1_ < 0, the nonlinear downstream wave height increases much faster than the linear ones as *ε*_2_ > 0 increases. On the contrary, if one fixes the second vortex *ε*_2_ > 0 and enlarges the strength of the first vortex *ε*_1_ < 0, the nonlinear downstream wave height goes up slowly and then decreases. Besides, the nonlinear wave height and linear wave height only coincide in the equal-strength case.

For co-rotating vortices, when the absolute values of the two point vortices are equal, the downstream wave height of the linear solution is zero at the point *d* = *mπL*_0_/2 (*m* odd), and the downstream wave height of the nonlinear solution is also zero at certain points. In this case, trapped waves, and even soliton-like waves are generated. At this time, the sign of the vortex pair plays an important role in the interferences of downstream waves due to two separate vortices, thus different phenomena occur for different signs of the pair. For the negative vortex pair, the nonlinear results first reach the minimum and maximum points. Similarly, for the positive vortex pair, the linear results first reach the minimum and maximum points. As for the amplitudes of the first crest due to two negative vortices, the nonlinear amplitudes are smaller than the linear amplitudes as the absolute value of vortex pair strength increases. For the amplitudes of the first crest due to two positive vortices, the nonlinear amplitude is bigger than linear amplitudes as the increasing strength of the vortex pair.

## Data Availability

Data and relevant code for this research work are stored in the Dryad Digital Repository: https://doi.org/10.5061/dryad.qnk98sfhj [42] and have been archived within the Zenodo repository: https://doi.org/10.5281/zenodo.5515469.

## Appendix A. The analytical expression of the wavenumber

In the appendix, we derive the analytical expression of *k*_0_ from equation (3.9). Since

A 1 $$\mathrm{artanh}x=\frac{1}{2}\ln\frac{1+x}{1-x},\;|x|<1,$$

(3.9) can be written as

A 2 $$\frac{2K_{0}}{1-\rho }=\frac{1-\rho \gamma^{2}}{\rho \gamma^{2}(1-\rho )}(k_{0}-K_{0})-\frac{k_{0}-K_{0}}{\rho \gamma^{2}F^{2}K_{0}}e^{2k_{0}\lambda },$$

where *K*_0_ is defined in (3.11). We denote

A 3 $$\omega =f\;(k)\;:=\frac{1-\rho \gamma^{2}}{\rho \gamma^{2}(1-\rho )}(k-K_{0})-\frac{k-K_{0}}{\rho \gamma^{2}F^{2}K_{0}}\;e^{2k\lambda },$$

thus equation (A 2) can be written as

A 4 $$\frac{2K_{0}}{1-\rho }=f\;(k_{0}).$$

Then using Lagrange–Bürmann formula (see [43, p. 14]), we obtain the inverse function of *f*(*k*)

A 5 $$k=K_{0}+\sum_{n=1}^{+\infty }\frac{1}{n!}\left[\lim_{k\to K_{0}}\frac{d^{n-1}}{dk^{n-1}}{\left(\frac{k-K_{0}}{f(k)-\omega_{0}}\right)}^{n}\right]{\left(\omega -\omega_{0}\right)}^{n},$$

where *ω*_0_ = *f*(*K*_0_) = 0. Finally supposing

A 6 $$\omega =\frac{2K_{0}}{1-\rho },$$

in (A 5) then the value of *k*_0_ is obtained

A 7 $$k_{0}=K_{0}+\sum_{n=1}^{+\infty }\frac{1}{n!}\left[\lim_{k\to K_{0}}\frac{d^{n-1}}{dk^{n-1}}{\left(\frac{k-K_{0}}{f(k)-\omega_{0}}\right)}^{n}\right]{\left(\frac{2}{F^{2}(1+\rho \gamma^{2})}\right)}^{n}.$$
